# The osteoarthritis inflammatory ecosystem: a node-edge-boundary-emergent framework toward precision stratification and therapeutic hypothesis generation

**DOI:** 10.3389/fmed.2026.1976217

**Published:** 2026-09-22

**Authors:** Zhiwen Zhang, Jiuyu Wei, Fuquan Du, Shaomeng Geng

**Affiliations:** Department of Orthopedics and Traumatology, The First Affiliated Hospital of Heilongjiang University of Chinese Medicine, Harbin, Heilongjiang, China

**Keywords:** biomarker, endotype, inflammatory ecosystem, macrophage, NEBE framework, osteoarthritis, precision medicine, synovitis

## Abstract

Osteoarthritis (OA) is a heterogeneous whole-joint disorder in which pain, structural progression and treatment response often diverge from radiographic severity. This mismatch has limited disease-modifying OA drug development and highlights the need for biologically informed patient stratification. Chronic low-grade inflammation contributes to OA progression, but it is embedded within mechanical loading, metabolic dysfunction, synovitis, osteochondral remodeling, cellular senescence and pain sensitization rather than acting as a single-cytokine process. Here, we propose a Node-Edge-Boundary-Emergent (NEBE) inflammatory ecosystem framework that organizes current OA evidence around cellular nodes, communication edges, boundary conditions and emergent disease states. We summarize evidence from human tissue studies, imaging-linked biomarker work, single-cell and spatial analyses, preclinical models and clinical trials. Mechanisms are organized around three translational processes: damage sensing, multicellular network amplification and failed inflammatory resolution. We then discuss candidate biological states, including inflammatory-dominant, metabolic, senescence-associated, osteochondral-remodeling and mechanical-structural OA, and map therapeutic strategies to the ecosystem level they primarily target. Prior failures of cytokine blockade and several disease-modifying approaches may reflect ecosystem mismatch involving the wrong target level, patient population, disease stage or endpoint rather than simple irrelevance of inflammation. Routine phenotyping and MRI may provide first-level stratification, biomarkers may support exploratory trial enrichment and high-cost omics should be reserved for discovery, validation and selected complex cases. Prospective human cohorts and biomarker-guided trials are required to determine whether NEBE-informed stratification improves target engagement, progression prediction or treatment selection beyond existing OA frameworks.

## 1 Introduction

### 1.1 Epidemiology and clinical burden of osteoarthritis

Osteoarthritis (OA) is the most common chronic joint disease and a major contributor to disability, pain, impaired mobility and health-care utilization worldwide (1–3). The global burden is increasing because of population aging, obesity, metabolic dysfunction, joint injury and high cumulative mechanical exposure. Recent burden estimates indicate that hundreds of millions of people are affected and that prevalence is projected to rise substantially in the coming decades (2). Knee, hip and hand OA account for most clinical burden, but spine and generalized phenotypes also contribute to disability and reduced quality of life. Current management remains largely symptomatic. Exercise, education, weight control and assistive measures are foundational; oral or topical non-steroidal anti-inflammatory drugs (NSAIDs), intra-articular corticosteroids and other analgesic approaches can reduce pain in selected patients; and joint replacement is effective for end-stage disease (4, 5). However, these interventions do not reliably restore cartilage, normalize synovial inflammation or prevent the progressive remodeling of the osteochondral unit. The absence of widely accepted disease-modifying OA drugs has stimulated renewed interest in mechanisms that connect mechanical stress, chronic low-grade inflammation, senescence, metabolic disturbance and repair failure. A practical clinical problem is that patients with similar radiographic severity may differ markedly in pain mechanisms, inflammatory activity, metabolic risk, structural progression and response to treatment. This heterogeneity has contributed to equivocal disease-modifying OA drug trials and makes average treatment effects difficult to interpret. For a medicine-oriented audience, the central question is therefore not only which mechanisms are biologically plausible, but which measurable patient states can guide diagnosis, enrichment, target engagement and therapeutic selection.

### 1.2 From cartilage wear to whole-joint inflammatory remodeling

OA was historically viewed as a passive wear-and-tear disease driven primarily by mechanical overload and articular cartilage erosion. This model remains partially correct because abnormal mechanical loading, joint injury and altered biomechanics are major initiators of tissue damage. Nevertheless, the cartilage-centric model is incomplete. OA is now regarded as a disease of the whole joint organ involving articular cartilage, synovium, meniscus, ligaments, infrapatellar fat pad, periarticular muscle and subchondral bone (3, 6, 7). Synovitis, vascular and neural remodeling, osteophyte formation and subchondral bone changes are not incidental findings; they contribute to pain and structural progression (8–11). Mechanical injury and matrix disruption release endogenous danger signals, including fibronectin and collagen fragments, alarmins, extracellular ATP, oxidized lipids, mitochondrial DNA and other DAMPs. These signals activate pattern-recognition receptors and stress-response pathways in synovial macrophages, fibroblasts and chondrocytes (12–18). The ensuing network of cytokines, chemokines, prostaglandins and proteases promotes matrix degradation and sustains a low-grade inflammatory state. Importantly, OA inflammation is weaker and more heterogeneous than autoimmune arthritis; it should therefore be interpreted as a disease-modifying component that interacts with mechanical, metabolic and aging-related factors rather than as the sole cause of OA.

### 1.3 Central hypothesis and scope of this review

Previous reviews have summarized inflammatory mediators, synovitis, macrophages, inflammasomes, metabolic-inflammation crosstalk, phenotype classification and regenerative therapies in OA. The major unresolved issue is how these components can be organized into a disease-sustaining structure that can be measured, modeled and therapeutically perturbed. The contribution of this review is not the recognition that inflammation participates in OA, but the organization of inflammatory mechanisms into a Node-Edge-Boundary-Emergent (NEBE) ecosystem framework. Rather than presenting a validated clinical classification system, NEBE is proposed as a testable conceptual framework that links cellular states, intercellular communication, boundary conditions and emergent disease manifestations. This organization aims to generate hypotheses for endotype assignment, biomarker development, trial enrichment and therapeutic matching.

Within this framework, OA inflammation is not a simple cytokine-driven response. It is an immune-metabolic ecosystem in which immune cells, stromal cells, chondrocytes, endothelial cells, osteochondral cells, senescent cells, matrix fragments and metabolic danger signals interact through network behavior. The same molecular pathway can have different consequences depending on cell type, joint compartment, disease stage and stimulus intensity. Disease-modifying strategies should therefore move from broad mediator suppression toward endotype-informed modulation of pathological node states, communication edges and boundary conditions (Figures 1–4).

**Figure 1:**
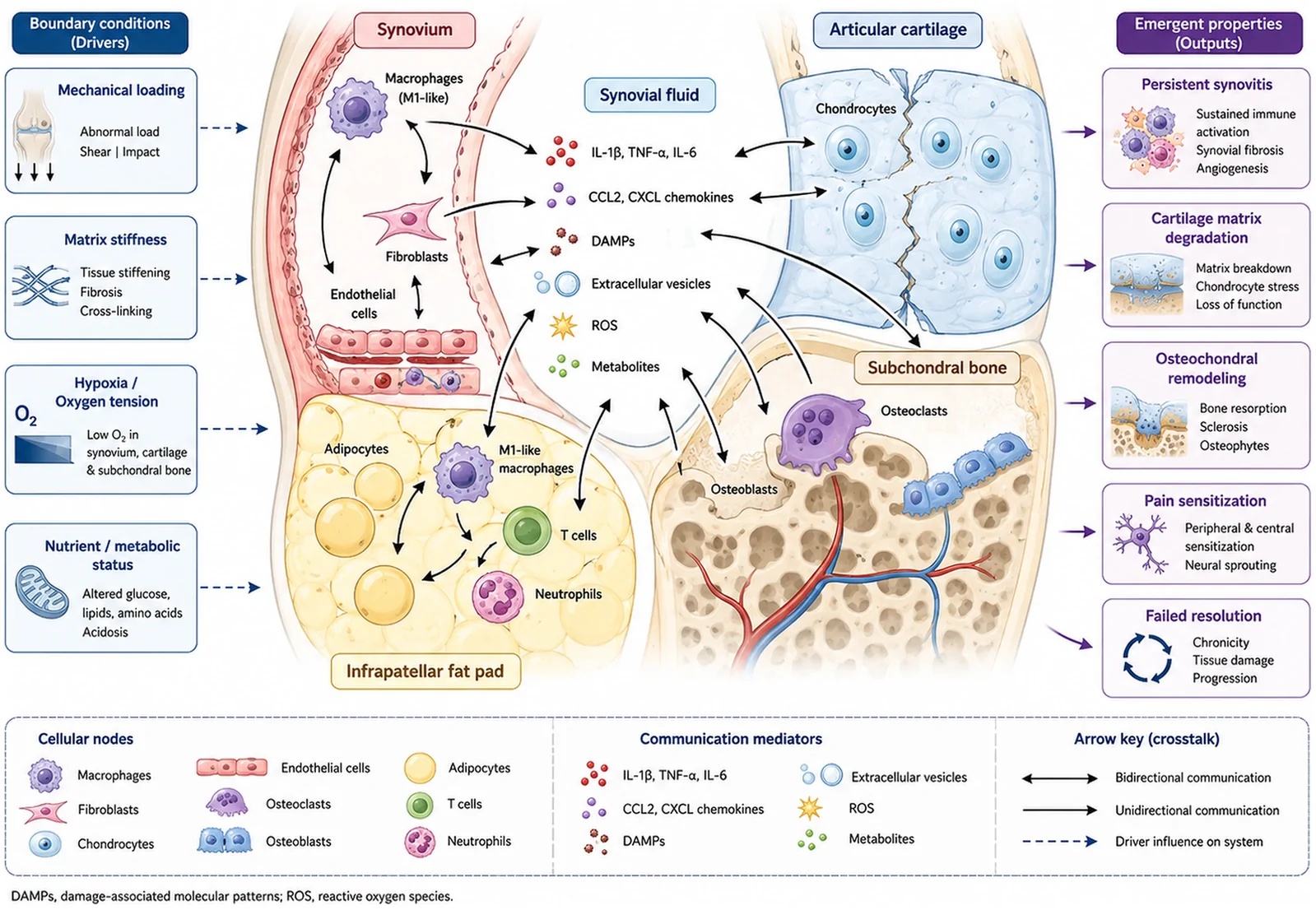
Layered NEBE ecosystem framework linking OA drivers, network states and clinical phenotypes. The figure organizes OA pathogenesis across four connected layers. External and host drivers include aging, obesity-associated metabolic stress, injury, malalignment and mechanical overload. The ecosystem layer includes macrophage-fibroblast activation, chondrocyte stress, endothelial and osteochondral remodeling, mitochondrial dysfunction, DAMP generation, immunometabolism and senescence. Clinical outputs include synovitis, pain sensitization, structural progression and treatment response. Intervention logic is mapped to the NEBE level targeted: edge-directed approaches modulate cytokine or chemokine communication, node-directed approaches reprogram macrophage, stromal or senescent-cell states, boundary-directed approaches modify loading or local exposure, and emergent-state strategies aim to resolve persistent synovitis, inflammatory memory or failed repair.

**Figure 2:**
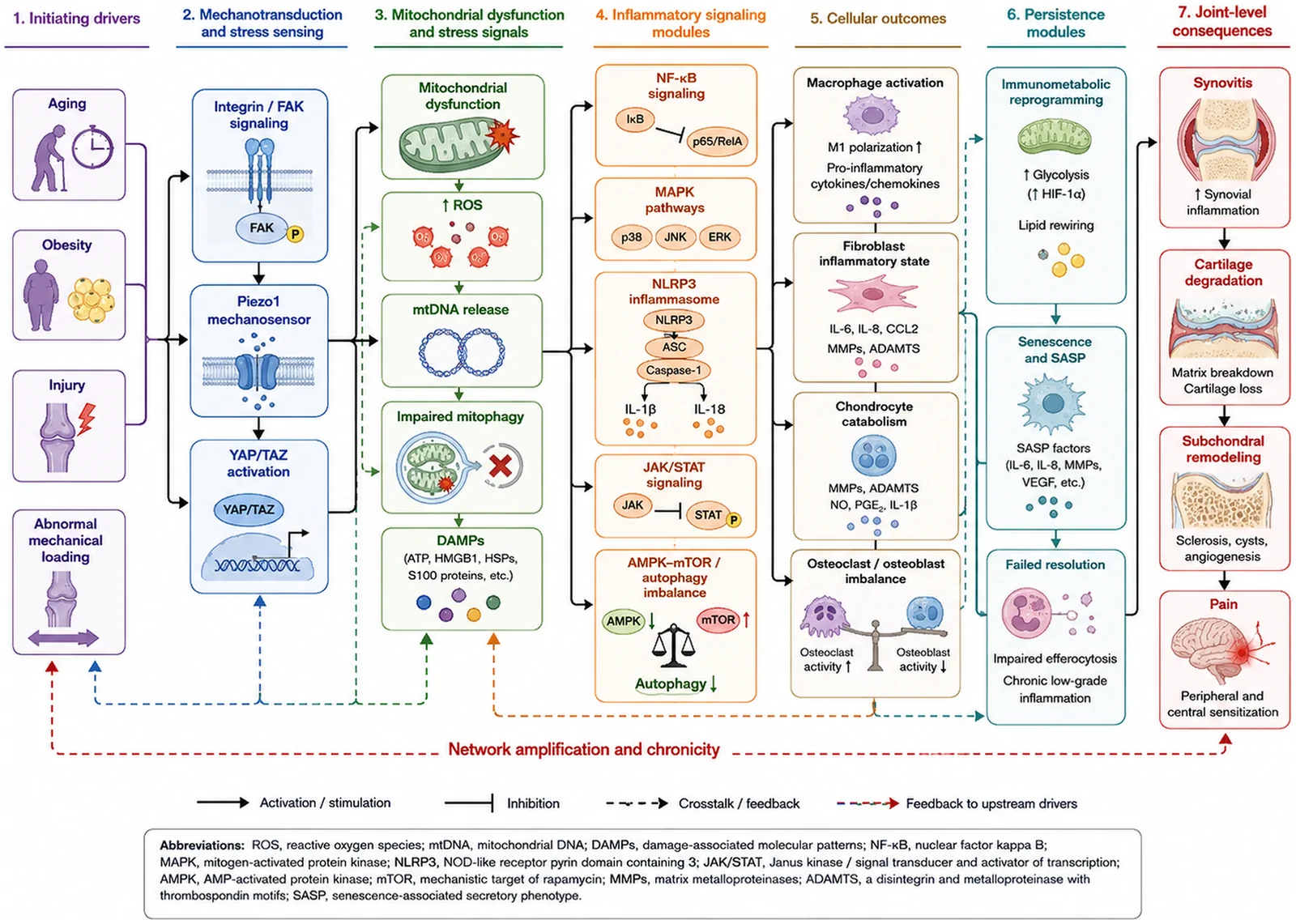
Integrated molecular network regulating OA inflammatory persistence. Mechanical stress activates integrin/FAK, Piezo channels and YAP/TAZ. Mitochondrial dysfunction generates ROS and DAMPs, which activate NF-kB, MAPK, JAK/STAT and inflammasome pathways. Autophagy failure, immunometabolic reprogramming, ferroptosis, pyroptosis and senescence reinforce chronic inflammation.

**Figure 3:**
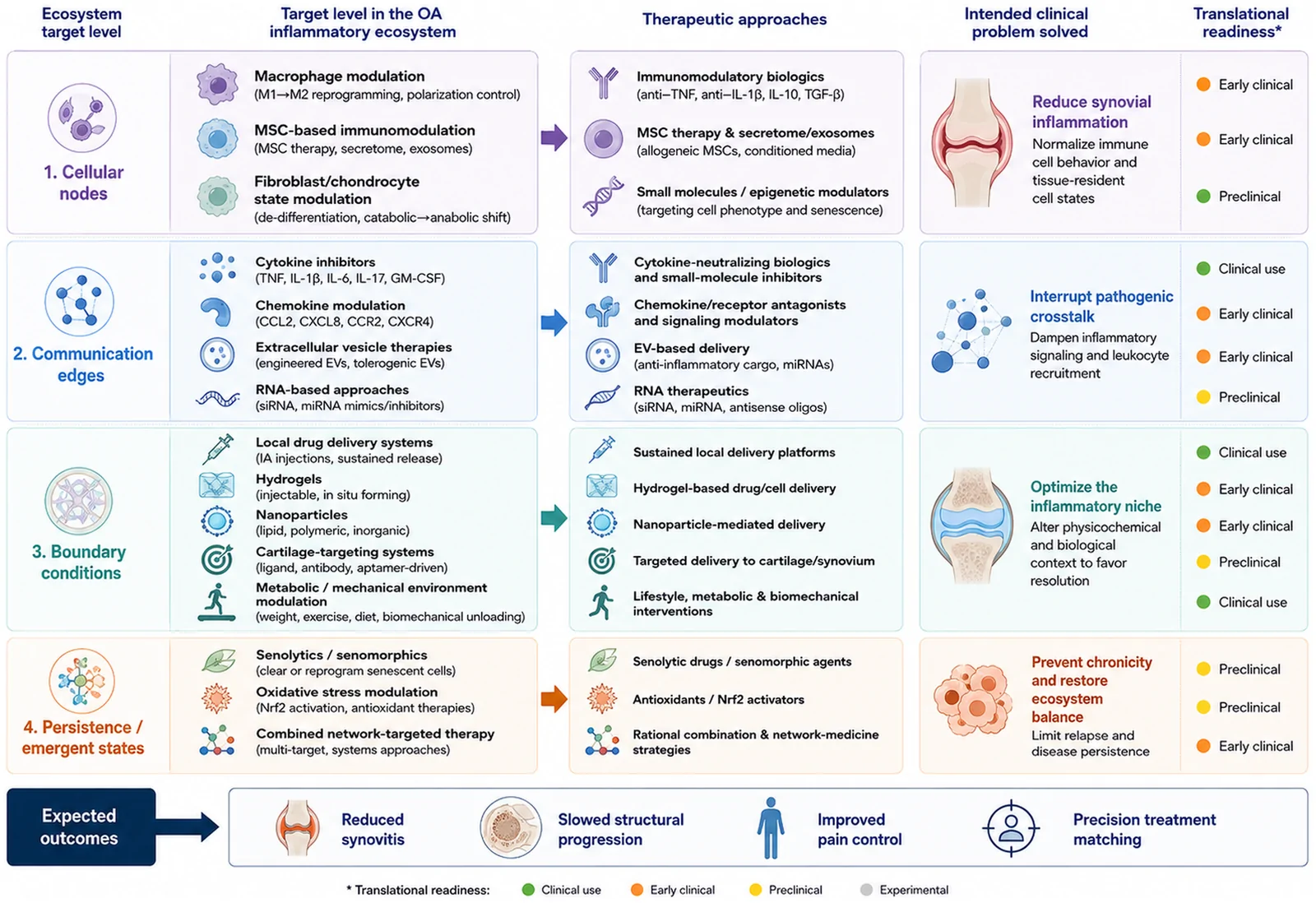
Therapeutic strategies mapped to NEBE target levels in the OA inflammatory ecosystem. Each therapeutic class is linked to the level of imbalance it primarily addresses. Edge-targeting therapies reduce cytokine or chemokine communication; node-targeting approaches reprogram macrophage, stromal or senescent-cell states; boundary-targeting systems such as hydrogels and nanoparticles improve local retention, tissue penetration and compartment access; and emergent-state strategies aim to resolve persistent synovitis, inflammatory memory or failed repair. Translational status should be interpreted cautiously: conventional anti-inflammatory drugs are clinically available mainly for symptom control, cytokine inhibitors have clinical trial evidence but are not established OA disease-modifying therapies, and extracellular vesicles, senescence-directed approaches, RNA therapeutics and nanomedicine remain preclinical or early translational.

**Figure 4:**
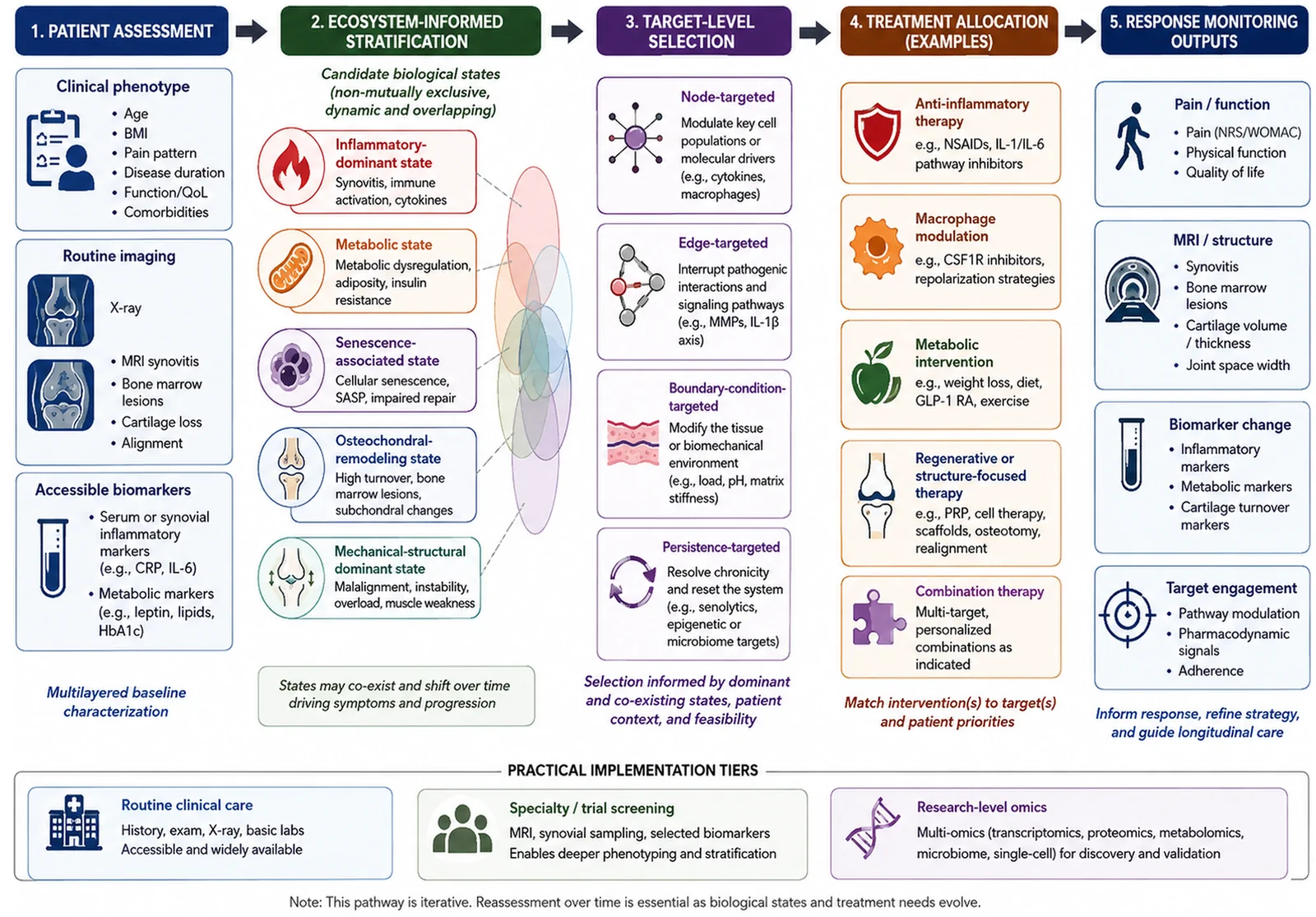
Ecosystem-to-clinic translation pathway. The workflow begins with symptomatic OA, routine clinical variables and imaging; proceeds to targeted biological stratification using synovitis, metabolic status, structural remodeling and candidate biomarkers; assigns a working biological state; selects an intervention according to target level; and monitors outcomes using matched clinical, imaging and molecular readouts. The figure separates routine clinical care, specialty or trial screening, and research or discovery tiers. Single-cell, spatial, proteomic and AI-supported analyses are positioned as discovery and trial-enrichment tools rather than routine requirements for every patient.

The ecosystem framework extends the “joint as an organ” concept by changing the explanatory question. The joint-as-organ concept defines where OA occurs by naming the tissues involved; the ecosystem framework explains how pathological information moves among these tissues, persists after the initiating trigger and becomes therapeutically actionable. Thus, the organ concept describes anatomical scope, whereas the ecosystem framework describes dynamics, feedback, boundary conditions and network behavior. This distinction matters clinically because anatomical breadth alone does not specify which tissue interaction sustains disease activity, which compartment should be sampled or which intervention level is most plausible. The ecosystem framework asks whether the dominant abnormality is a cellular node state, a communication edge, a boundary condition or an emergent state, thereby translating anatomical complexity into measurable questions for biomarkers, imaging, trial enrichment and therapeutic selection.

Working definition. We define the OA inflammatory ecosystem as a spatially organized, functionally interconnected network captured by the NEBE model: (1) cellular nodes, including macrophages, fibroblasts, chondrocytes, endothelial cells, osteochondral cells and adaptive or innate immune cells; (2) communication edges, including cytokines, chemokines, DAMPs, metabolites, extracellular vesicles and direct cell-contact signals; (3) boundary conditions, including matrix stiffness, oxygen tension, nutrient availability, synovial-fluid composition, local drug exposure and mechanical loading; and (4) emergent properties, including synovitis persistence, chondrocyte state transitions, osteochondral remodeling, pain sensitization and failed resolution of repair. This framework shifts the therapeutic question from “which single molecule drives OA?” to “which node, edge, boundary condition or emergent state is most amenable to intervention in a given patient?”

Practical rules for NEBE assignment. Components are assigned according to their primary biological role rather than their molecular identity alone. A node is a disease-relevant cell population or cellular state, such as macrophage activation, fibroblast activation or chondrocyte senescence. An edge is a communication signal that connects biological components, such as IL-1beta, TNF-alpha, IL-6, CCL2, DAMPs, adipokines, metabolites, extracellular vesicle cargo or cell-contact signaling. A boundary condition is a local or systemic constraint that shapes ecosystem behavior, such as abnormal loading, malalignment, matrix stiffness, hypoxia, obesity-associated metabolic stress or limited intra-articular drug exposure. An emergent state is a system-level pattern generated by interactions among multiple nodes, edges and boundaries, such as persistent synovitis, pain sensitization, osteochondral remodeling, inflammatory memory or failed repair. A factor may influence more than one level, but classification in this review is based on its dominant role in the specific biological or therapeutic context being discussed.

Emergent properties require explicit clarification. In this review, emergent does not simply mean that several pathological features occur together. It means that persistence of synovitis, instability of chondrocyte states, osteochondral remodeling, pain sensitization and failed repair cannot be predicted from the activity of any single node or edge alone. For example, sustained synovitis may arise from reciprocal macrophage-fibroblast feedback, metabolic-epigenetic reinforcement, matrix-DAMP generation and altered tissue mechanics that maintain inflammation after the initiating trigger has diminished. This non-additive behavior is the main reason why the ecosystem framework implies different therapeutic strategies from single-target cytokine frameworks.

Relationship to recent reviews. Recent reviews have examined inflammaging and immunosenescence in OA (14), inflammation-metabolism crosstalk (15), systems-level failure of inflammatory regulation (16) and phenotype-based precision medicine (17). The present manuscript differs by using these related literatures to define a structured NEBE model that links mechanisms to target-level decision logic. Existing whole-joint models identify the anatomical scope of OA; phenotype and endotype frameworks describe patterns of heterogeneity; and systems-biology approaches characterize molecular networks. NEBE does not replace these frameworks. Its added value is to connect biological components to three practical research decisions: which patients should be enriched for a mechanism-matched trial, which ecosystem level should be targeted and which clinical, imaging or molecular endpoint best matches the intervention mechanism. This distinction is summarized in Table 1 and motivates the validation logic in Sections 5.9 and 5.10.

**Table 1:** Comparison of the NEBE framework with recent OA review frameworks.

Framework or review focus	Primary focus	Remaining gap	NEBE contribution
Inflammaging/immunosenescence (14)	Aging-related immune remodeling	Limited target-level workflow	Treats aging and senescence as node, boundary and emergent-state abnormalities.
Inflammation-metabolism crosstalk (15)	Metabolic-inflammatory pathways and therapies	Not primarily a stratification model	Maps metabolic stress as a boundary condition and node-state driver.
Systems failure of inflammatory regulation (16)	Regulatory failure and resolution pathways	Selected regulatory axes	Extends systems logic into a broader clinical node-edge-boundary workflow.
OA phenotypes and precision medicine (17)	Clinical, structural and molecular phenotypes	Weak link between phenotype labels and intervention level	Converts phenotypes into candidate ecosystem states, trial-enrichment criteria, target-level selection and endpoint matching.
Current NEBE framework	Testable target-level organizing framework of OA inflammation	Requires prospective validation	Provides endotype-assignment logic, ecosystem-mismatch logic and falsifiable predictions for trial design.

Testability of the framework is essential. The ecosystem framework is not intended as a metaphor for complexity; it generates predictions regarding network-level biomarkers, multi-node disease progression and endotype-matched therapeutic response that can be evaluated in longitudinal cohorts and stratified trials. Scope and contribution to the field. This review is intended to contribute a translational organization of existing OA inflammatory knowledge rather than a claim of new primary discovery. Its clinical relevance depends on whether future human studies show that NEBE-informed classification improves prediction or treatment matching beyond conventional radiographic and symptom-based approaches.

### 1.4 Search strategy for this narrative review

A targeted narrative literature strategy was used to identify mechanistic, translational and clinical studies relevant to OA inflammatory microenvironments. PubMed/MEDLINE, Web of Science and major journal databases were searched from database inception to 5 September 2026 using combinations of the following terms: osteoarthritis, synovitis, inflammatory microenvironment, macrophage, fibroblast, chondrocyte, single-cell RNA sequencing, spatial transcriptomics, NF-kB, NLRP3 inflammasome, MAPK, JAK/STAT, mechanotransduction, Piezo, YAP/TAZ, mitochondria, autophagy, immunometabolism, inflammaging, immunosenescence, senescence, ferroptosis, extracellular vesicles, nanomedicine, endotype, evidence hierarchy, biomarker, clinical trial and precision medicine.

Original studies, randomized trials, guidelines and high-quality reviews were prioritized. Landmark studies before 2010 were retained when they provided foundational mechanistic or clinical concepts. Studies were included when they investigated human OA tissues, synovial fluid, imaging cohorts, clinical trials, or mechanistic studies directly relevant to inflammatory regulation, cellular states, biomarkers or therapeutic translation. Studies unrelated to OA, inaccessible full-text reports, non-English articles without accessible English abstracts or full texts, and purely descriptive reports without direct mechanistic or translational relevance were excluded.

Because this manuscript is a narrative review rather than a systematic review, study selection was not performed as a PRISMA-based systematic review. Evidence interpretation and grading were based on expert narrative assessment. Two authors independently evaluated the maturity of evidence for the major cellular and molecular modules, and disagreements were resolved by discussion and consensus. The grading system was intended to clarify the evidentiary boundary between human association, preclinical mechanism and clinical targetability rather than to provide formal GRADE certainty.

### 1.5 Early validation logic and testable predictions of the ecosystem framework

Because this review uses ecosystem language as an organizing framework rather than a decorative metaphor, its claims must be testable. Three validation questions follow directly from the framework and are expanded in Section 5.10. First, in synovitis-enriched OA, does node-level modulation of macrophage or stromal-cell states outperform single-edge cytokine blockade when target engagement is confirmed? Second, do patients with multi-compartment network burden progress faster than patients with isolated abnormalities after adjustment for conventional risk factors? Third, do multivariable network-based models improve prognosis or treatment-response prediction beyond single-marker approaches with clinically meaningful gains in calibration, discrimination or decision-curve benefit? These predictions foreground the proposed sequence from ecosystem assessment to endotype assignment and therapy selection.

### 1.6 Clinical implications of ecosystem-guided OA management

Box 1: Conceptual evolution of OA pathogenesis. Conceptual evolution of OA pathogenesis Classical view: OA as a mechanical degenerative disease dominated by cartilage wear, mechanical overload and extracellular matrix loss. Current view: OA as a whole-joint disorder involving cartilage, synovium, subchondral bone, meniscus, ligaments, vasculature and sensory nerves. Emerging view: OA as an immune-metabolic ecosystem disorder in which mechanical stress, inflammatory communication, metabolic adaptation, senescence and failed repair reinforce each other. Note: This box summarizes conceptual evolution; supporting references are discussed in the relevant text sections. The clinical value of the ecosystem framework is that it translates biological complexity into a staged decision pathway rather than requiring every patient to undergo expensive molecular profiling. In routine care, the first step remains clinical phenotyping: age, body mass index, metabolic comorbidity, disease duration, pain pattern, alignment, prior injury, joint distribution and radiographic severity. These variables do not define molecular endotypes by themselves, but they identify the likely boundary conditions that shape the joint ecosystem. The second step is biological stratification using tools already available in many specialty settings. MRI can identify synovitis, effusion, bone marrow lesions, meniscal damage and osteochondral remodeling. Serum or synovial-fluid markers may provide additional information about inflammation, tissue turnover or metabolic stress, although most are not yet validated for routine treatment selection. When these measures point in the same direction, they can define a clinically useful working endotype. For example, painful knee OA with effusion-synovitis and inflammatory chemokines is more plausibly inflammatory-dominant than radiographic OA without synovial activation. The third step is therapeutic matching. Inflammatory-dominant OA may be enriched for trials of immune modulation or macrophage-state regulation; metabolic OA may require weight loss, metabolic risk control and mitochondrial or AMPK-related interventions; senescence-associated OA remains a research endotype that may eventually support senomorphic or senolytic strategies; osteochondral-remodeling OA requires attention to bone marrow lesions, angiogenesis, subchondral bone and pain mechanisms; and mechanical-structural dominant OA requires biomechanical correction, rehabilitation, bracing or surgical approaches when appropriate. This tiered workflow also protects the framework from becoming clinically unrealistic. Single-cell RNA sequencing, spatial transcriptomics and multi-omics are essential for discovery and trial enrichment, but they are not required for every patient in routine practice. Their immediate role is to identify cellular nodes and communication edges that can later be approximated by practical imaging or biomarker panels. The central test is whether such stratification improves progression prediction, target engagement and treatment response compared with radiographic grade alone.

### 1.7 From ecosystem states to clinically actionable endotypes

The ecosystem framework becomes clinically useful only if it translates biological heterogeneity into actionable categories. At present, these categories should be interpreted as candidate biological states rather than fully validated clinical endotypes. Their clinical utility requires confirmation through longitudinal cohorts that integrate molecular, imaging and outcome data, and through interventional studies showing that state-informed treatment selection improves target engagement or clinical response. Here, the term endotype is used as a pragmatic shorthand for a dominant ecosystem state, not as a claim that stable diagnostic subtypes have already been established. Because these states may coexist within the same patient and evolve over time, ecosystem-based classification should be regarded as probabilistic and time-dependent rather than categorical or permanent. A radiographic grade captures structural severity; it does not identify whether the dominant network process is synovitis, metabolic stress, senescence, osteochondral remodeling or mechanical overload.

Clinically actionable endotyping should begin by identifying which ecosystem layer is most abnormal. A node abnormality, such as macrophage-fibroblast activation, supports node-reprogramming strategies. A dominant communication edge, such as a cytokine or chemokine signature, supports mediator-directed therapy but does not correct mechanical or metabolic boundary conditions. A boundary abnormality, such as malalignment, obesity-associated metabolic stress, hypoxia or poor tissue penetration, requires correction of the upstream context rather than molecular inhibition alone.

This logic changes trial design. Traditional OA trials enroll patients by symptoms and radiographic criteria, then estimate an average treatment effect in a biologically mixed population. Ecosystem-guided trials should enrich for the target-relevant state: synovitis, effusion, inflammatory biomarkers or macrophage-related signatures for immune-modulating therapies; bone marrow lesions or subchondral remodeling markers for osteochondral therapies; and metabolic or loading markers for boundary-directed interventions. This reduces biological dilution and the risk of false-negative trials.

A staged workflow is most realistic. Routine clinical features and radiographs provide first-level sorting; MRI adds synovitis, effusion, bone marrow lesions and cartilage defects; serum or synovial-fluid biomarkers refine inflammatory, metabolic or tissue-turnover states; and single-cell, spatial, proteomic or AI-supported models remain research tools for translating network states into simpler decision rules. Inflammatory-dominant OA may currently represent one of the most feasible candidate states for exploratory trial enrichment because synovitis and effusion can be assessed using imaging and fluid-based approaches. However, these features should not be interpreted as validated predictors of treatment response. Prospective studies are still required to determine whether inflammatory markers can reliably guide patient selection, target engagement assessment and therapeutic benefit. Metabolic OA is emerging; senescence-associated OA remains experimental; and mechanical-structural OA is already actionable for biomechanical management.

## 2 Cellular architecture of the OA inflammatory ecosystem

### 2.1 Synovial inflammatory niche

Synovitis is among the most reproducible inflammatory features of OA and is associated with pain, effusion and structural progression (8, 9). OA synovium commonly exhibits lining-layer hyperplasia, macrophage infiltration, fibroblast activation, vascular changes and variable lymphocyte accumulation. Activated synovial cells produce IL-1beta, TNF-alpha, IL-6, CCL2, CXCL chemokines, prostaglandins, nitric oxide and matrix-degrading enzymes (8, 9, 12–19). These mediators stimulate chondrocytes to increase MMP and ADAMTS expression, thereby accelerating collagen II and aggrecan degradation (20, 21). Synovitis should not be regarded as a passive consequence of cartilage loss. Damaged cartilage and meniscal tissues generate DAMPs that activate innate immune pathways, while synovial inflammation feeds back onto cartilage and subchondral bone. This bidirectional communication explains why synovitis can be present in early OA and why imaging-defined synovial inflammation may identify patients with more active disease. However, synovial inflammation is heterogeneous; anti-inflammatory treatment is likely to benefit only the subgroup in which synovitis is a dominant active mechanism rather than a late secondary response.

### 2.2 Macrophage functional states

Macrophages are central immune regulators in OA joints. Classical M1/M2 terminology remains useful as a simplified language, but human OA macrophages occupy a continuum of context-dependent functional states rather than two fixed phenotypes (19, 22, 23). Inflammatory macrophage states produce IL-1beta, TNF-alpha, IL-6, nitric oxide, ROS and chemokines. Resolving or reparative states participate in efferocytosis, matrix remodeling, IL-10 and TGF-beta-related repair programs. Excessively prolonged repair-associated activation can also promote fibrosis, indicating that macrophage biology cannot be reduced to “bad M1” and “good M2” categories. Macrophages function as hubs connecting synovial inflammation, chondrocyte catabolism, fibroblast activation and osteochondral remodeling. Single-cell studies increasingly indicate that macrophage populations differ in inflammatory cytokine expression, antigen presentation, interferon response, lipid metabolism and pain-associated signatures (24, 25). Therefore, therapeutic strategies should aim to reprogram disease-associated macrophage states rather than deplete macrophages globally. Such reprogramming may be achieved through metabolic regulators, biomaterial cues, extracellular vesicle cargo or local cytokine balance, but human markers of target engagement remain insufficiently validated.

### 2.3 Synovial fibroblasts as inflammatory amplifiers

Synovial fibroblasts are increasingly recognized as active organizers of inflammation rather than structural bystanders. In OA, activated fibroblasts produce IL-6, CCL2, CXCL8, CXCL12, growth factors and matrix-remodeling enzymes. These mediators recruit and activate immune cells, influence vascular remodeling and propagate synovitis. Although fibroblast biology is better defined in rheumatoid arthritis, OA single-cell analysis has also revealed heterogeneity among fibroblast and stromal populations (25). Fibroblast-macrophage interactions may be especially important. Macrophage-derived IL-1beta and TNF-alpha can increase fibroblast chemokine production; fibroblast-derived CCL2 and IL-6 can sustain macrophage recruitment and activation. This reciprocity creates an inflammatory niche that can persist even when the initial tissue injury is no longer dominant. Future studies should distinguish inflammatory fibroblast subsets, matrix-producing fibroblasts and vascular-associated stromal cells in OA tissue, because bulk synovial suppression may be less effective than targeting disease-associated stromal networks.

### 2.4 Chondrocytes as immune-responsive regulators

Chondrocytes maintain cartilage matrix homeostasis under physiological conditions, but they also function as immune-responsive cells. Under mechanical overload, cytokine exposure, oxidative stress and matrix disruption, chondrocytes acquire catabolic, hypertrophic-like, inflammatory, apoptotic or senescent states (18, 24, 26). IL-1beta and TNF-alpha activate NF-kB and MAPK signaling, increasing MMP-13, ADAMTS-4/5, cyclooxygenase-2 and inducible nitric oxide synthase. The resulting matrix degradation releases additional fragments that activate innate immune receptors, forming a cartilage-synovium feedback loop. Single-cell RNA sequencing has identified multiple OA-associated chondrocyte states, including populations linked to matrix organization, hypertrophic differentiation, inflammatory signaling and cartilage degradation (24). These findings suggest that cartilage degeneration reflects pathological state transitions rather than uniform chondrocyte failure. Senescent chondrocytes are particularly relevant because they release SASP factors such as IL-6, IL-8, chemokines and MMPs, thereby propagating inflammation and impairing matrix repair (27–30).

### 2.5 Adaptive and innate immune amplifiers

OA is not a classical autoimmune disease, but adaptive immune cells can modulate the inflammatory microenvironment. CD4 + and CD8+ T cells, Th17-like populations and regulatory T cells have been detected in OA tissues. IL-17A can stimulate chondrocytes, macrophages and fibroblasts to increase inflammatory mediators and matrix-degrading enzymes, whereas regulatory T-cell programs may restrain excessive inflammation. Compared with rheumatoid arthritis, adaptive immunity in OA is likely to be a disease modifier rather than a primary antigen-driven initiating mechanism. Innate immune amplification also involves neutrophils, mast cells and complement. Neutrophils and NETs may contribute through ROS, proteases, DNA scaffolds and histones that activate Toll-like receptor and inflammasome pathways; however, human OA evidence remains limited, and NETs should be interpreted as an active area of investigation rather than an established clinical driver. Mast cells may influence vascular permeability, pain and immune recruitment, whereas complement activation products such as C3a, C5a and membrane attack complex have stronger support from human samples and experimental models (31). These modules broaden OA inflammation beyond macrophage- and cytokine-focused frameworks, but their therapeutic relevance is likely endotype- and stage-dependent.

### 2.6 Single-cell and spatial perspectives on cellular crosstalk

Single-cell studies have changed how the OA inflammatory microenvironment is interpreted. Earlier models often described OA tissues using broad categories such as inflamed synovium, damaged cartilage or sclerotic bone. In contrast, single-cell data indicate that each compartment contains disease-associated cell states that cannot be captured by bulk measurement. In cartilage, OA-associated chondrocytes include populations enriched for matrix organization, stress response, inflammatory signaling, hypertrophic differentiation and degenerative transcripts (24). In synovium, fibroblast, macrophage, endothelial and lymphoid populations show heterogeneity that may explain why synovitis varies substantially among patients (25). The next conceptual advance is spatial organization. A cell state becomes clinically meaningful only if its anatomical location and communication partners are understood. A macrophage population adjacent to activated fibroblasts in vascularized synovium may exert a different effect from one located near the osteochondral interface. Similarly, senescent superficial-zone chondrocytes may release mediators into synovial fluid, whereas deep-zone chondrocytes may communicate more strongly with subchondral bone. Spatial transcriptomics, multiplex imaging and ligand-receptor analysis can transform single-cell catalogues into maps of actionable inflammatory niches.

### 2.7 Endothelial cells, angiogenesis and osteochondral communication

Angiogenesis and neurovascular remodeling link inflammation to pain and structural progression. Mature articular cartilage is avascular, but OA is characterized by vascular invasion at the osteochondral junction, synovial vascular proliferation and sensory nerve growth (11). VEGF, PDGF-BB, inflammatory cytokines and hypoxia-related signals promote endothelial activation and new vessel formation. These vessels provide routes for immune-cell recruitment and are often accompanied by sensory innervation, contributing to pain sensitization. The cartilage-subchondral bone unit functions as an integrated pathological compartment. Altered osteoblast and osteoclast activity, increased bone turnover, RANK/RANKL/OPG imbalance, aberrant TGF-beta and Wnt signaling, angiogenesis and marrow changes influence cartilage homeostasis (10, 32). Osteoclast-lineage-derived PDGF-BB can stimulate angiogenesis in subchondral bone and contribute to OA development (32). These findings explain why cartilage-only therapies often fail to achieve durable disease modification: OA involves communication across the osteochondral unit, synovium and vascularized tissues.

### 2.8 Infrapatellar fat pad, adipokines and neuroimmune pain signaling

The infrapatellar fat pad is increasingly viewed as an immunometabolic tissue within the knee joint. It contains adipocytes, macrophages, stromal cells, vascular cells and sensory nerve fibers, and it releases adipokines, cytokines, lipid mediators and free fatty acids that can influence synovium and cartilage. In obesity-associated OA, systemic metabolic inflammation may converge with local fat-pad inflammation, providing a mechanism by which excess adiposity increases OA risk beyond mechanical loading alone. Leptin, adiponectin, resistin and visfatin have all been investigated as mediators linking adipose tissue, inflammation and cartilage catabolism, although their effects vary by concentration, receptor context and cell type. Pain is also an integral part of the inflammatory microenvironment rather than a purely mechanical consequence of cartilage loss. Cartilage itself is aneural, but synovium, subchondral bone, meniscus, capsule and periarticular tissues are innervated. Synovitis, angiogenesis and sensory nerve ingrowth can increase nociceptive signaling, while NGF, prostaglandins, bradykinin, chemokines and cytokines sensitize peripheral nerves. Bone marrow lesions and osteochondral vascular invasion provide additional pain-generating compartments. Therefore, therapies that restore inflammatory balance may improve pain even when cartilage structure changes slowly, whereas structure-directed therapies may fail to relieve pain if neuroimmune sensitization persists.

### 2.9 Summary of cellular-network logic

Taken together, OA cellular pathology is organized as a network. Synovial macrophages and fibroblasts generate a local inflammatory niche; chondrocytes respond by shifting toward catabolic, hypertrophic-like and senescent states; damaged matrix feeds back into innate immune activation; endothelial and osteochondral cells promote angiogenesis and nerve growth; adipose and metabolic tissues add systemic inflammatory inputs; and adaptive immune cells, neutrophils, mast cells and complement modify the intensity and persistence of inflammation. This network logic is central for therapy: a patient with active synovitis, obesity and bone marrow lesions may require a different intervention from a patient with mechanically localized cartilage loss and minimal inflammatory activity. Returning to the operational definition, synovial macrophages, fibroblasts, chondrocytes, endothelial cells, fat-pad cells and osteochondral cells represent cellular nodes. Cytokines, chemokines, DAMPs, adipokines, lipid mediators, extracellular vesicles and cell-contact signals represent communication edges. Matrix stiffness, oxygen tension, vascular access, nutrient availability, adiposity and abnormal loading represent boundary conditions. Synovitis persistence, chondrocyte-state instability, failed resolution, osteochondral remodeling and pain amplification represent emergent properties. This organizational logic explains why modulating a single edge, such as IL-1beta, without altering disease-associated node states or boundary conditions is unlikely to restore system-level homeostasis.

### 2.10 Temporal evolution of the OA inflammatory ecosystem

The ecosystem framework is spatial and temporal. OA progression can be conceptualized as a transition from adaptive stress responses to amplification, persistence and structural irreversibility. These phases overlap within the same joint, but the temporal view explains why the same therapy may differ in efficacy across early, symptomatic and advanced disease. During initiation, abnormal loading, injury, aging or metabolic stress alters boundary conditions such as matrix stiffness, mechanical strain and tissue oxygenation. Chondrocytes and synovial cells sense these changes through integrins, Piezo channels and cytoskeletal pathways, while damaged matrix releases DAMPs. Inflammation at this stage may still support debris clearance and repair, so the objective is prevention of maladaptive conversion rather than maximal immune suppression. During amplification and persistence, reciprocal macrophage-fibroblast-chondrocyte communication becomes more important than the initiating trigger. Cytokines, chemokines, matrix fragments, EV cargo, mitochondrial stress and SASP-like mediators create feedback loops that sustain synovitis, pain fluctuation and matrix degradation. This phase provides the strongest rationale for endotype-matched node modulation or boundary correction rather than isolated mediator blockade. In advanced phases, osteochondral remodeling, fibrosis, angiogenesis, nerve ingrowth, cartilage loss and biomechanical instability may dominate. Anti-inflammatory therapy alone is unlikely to restore system-level homeostasis when boundary conditions have become fixed. Precision therapy therefore requires the right target at a stage in which the ecosystem remains modifiable.

## 3 Molecular mechanisms regulating OA inflammatory persistence

This section is organized around three ecosystem processes rather than an exhaustive pathway catalogue: damage sensing, network amplification and failed inflammatory resolution. Mechanotransduction and DAMP generation convert tissue injury into biological signals; NF-kB, MAPK, JAK/STAT and inflammasome pathways amplify communication among cells; and mitochondrial dysfunction, altered autophagy, immunometabolism and senescence help maintain chronic inflammatory memory.

### 3.1 NF-kB signaling as a multicellular inflammatory hub

NF-kB is treated here as a communication amplifier rather than a stand-alone pathway. IL-1, TNF, Toll-like receptor ligands, ROS, integrin signals and matrix-derived DAMPs converge on IKK-dependent IkB degradation and RelA/p50 nuclear translocation (33–35). In chondrocytes this increases inflammatory and catabolic mediators; in macrophages it supports cytokine and chemokine release; and in fibroblasts it reinforces leukocyte recruitment and synovitis. NF-kB also primes inflammasome components and contributes to SASP regulation, linking damage sensing to inflammatory memory. These broad functions make local or cell-selective modulation more plausible than systemic inhibition.

### 3.2 NLRP3 inflammasome and pyroptotic amplification

The NLRP3 inflammasome links cellular stress to IL-1 family cytokine maturation and pyroptotic amplification. NF-kB-dependent priming can increase NLRP3 and pro-IL-1beta, while ATP, mitochondrial ROS, ionic imbalance, oxidized lipids or mitochondrial DNA provide activation signals (36, 37). In OA, inflammasome-associated activity has been reported in macrophages, synovial cells and chondrocytes, but stress-induced cartilage degradation can also occur independently of NLRP3 in selected human OA and mouse-model settings (38). NLRP3 is therefore best interpreted as a context-dependent edge amplifier in inflammasome-active endotypes rather than as a universal disease driver.

### 3.3 MAPK and JAK/STAT signaling

MAPK and JAK/STAT signaling are network-amplification modules. ERK, JNK and p38 MAPK integrate mechanical strain, cytokines, matrix fragments and oxidative stress, converting stress inputs into AP-1-related transcriptional programs that regulate inflammatory mediators, MMPs, apoptosis and hypertrophic-like differentiation (39). IL-6-family ligands activate gp130-associated JAKs and STAT3, supporting inflammatory and catabolic programs in selected OA cells (40, 41). Their clinical relevance depends on endotype and stage because OA inflammation is lower-grade, localized and less cytokine-dominant than autoimmune arthritis.

### 3.4 Mechanotransduction: integrins, piezo channels and YAP/TAZ

Mechanical signals are translated through integrins, focal adhesion kinase, cytoskeletal tension, Piezo channels and Hippo pathway effectors. These mechanisms are boundary sensors: abnormal loading changes matrix stiffness, ion flux, YAP/TAZ activity and MAPK signaling, thereby altering chondrocyte state and inflammatory responsiveness (40–44). YAP/TAZ effects remain context-dependent; physiological loading may support matrix homeostasis, whereas excessive stiffness or abnormal loading can cooperate with inflammatory signals. Thus, mechanotransduction converts boundary conditions into cell-state changes rather than acting as a uniformly pro-inflammatory pathway.

### 3.5 Oxidative stress, mitochondrial dysfunction and mitophagy

Mitochondria form a convergence point for mechanical injury, aging, metabolic stress and inflammation. Dysfunctional mitochondria generate ROS, alter ATP production and release danger molecules that activate NF-kB, MAPK and inflammasome-associated pathways; cytokines further damage mitochondria, creating feed-forward amplification (45). Mitophagy limits ROS and mitochondrial danger-signal accumulation, and metformin can enhance SIRT3-PINK1/Parkin-dependent mitophagy under inflammatory stimulation (46). However, mitophagy is context-dependent and may be maladaptive in severe metabolic-toxic settings (47). The therapeutic target is physiological quality-control flux, not maximal autophagy activation.

### 3.6 AMPK-mTOR axis, autophagy and immunometabolic signaling

Autophagy and immunometabolism connect boundary conditions with node state. Age-related autophagy decline is linked to chondrocyte death and OA, while cartilage-specific mTOR deletion enhances autophagy and protects against experimental OA (48, 49). Inflammatory macrophage states often rely on glycolysis and lactate production, whereas resolving states depend more on oxidative phosphorylation and fatty-acid metabolism (50, 51). In chondrocytes, LDHA-mediated glycolytic reprogramming can increase ROS and inflammatory-catabolic mediators, and cartilage-specific LDHA deletion reduced OA severity in mice (52). These findings support metabolic regulation as a research priority, but human clinical validation of metabolite-based biomarkers or interventions remains incomplete.

### 3.7 Epigenetic regulation and non-coding RNAs

Epigenetic and non-coding RNA mechanisms can stabilize OA cell states by regulating cytokine expression, matrix metabolism, senescence and intercellular signaling (53, 54). For this framework, their main relevance is transcriptional memory and cell-state persistence; most individual miRNA, lncRNA or circRNA targets remain preclinical rather than clinically actionable.

### 3.8 Regulated cell death: pyroptosis and ferroptosis

Regulated cell death modifies OA inflammation by changing tissue composition and releasing inflammatory contents. Pyroptosis connects inflammasome activation with IL-1 family cytokine release, whereas ferroptosis reflects iron-dependent lipid peroxidation and antioxidant failure (55–59). These mechanisms are best viewed as modifiers of mitochondrial stress and failed resolution rather than stand-alone explanations for OA progression; human evidence remains less mature than for synovitis, macrophage activation, matrix catabolism or senescence.

### 3.9 Cellular senescence and inflammatory memory

Cellular senescence converts transient injury into inflammatory memory. Senescent chondrocytes and stromal cells exhibit stable cell-cycle arrest, altered metabolism, DNA-damage responses, apoptosis resistance and SASP secretion that can activate neighboring chondrocytes, macrophages and fibroblasts. Local clearance of senescent cells reduced post-traumatic OA in experimental models (27), but senescence-targeted therapy must distinguish pathological chronic senescence from transient repair-associated senescence, and proposed biomarkers such as p16INK4a or DPP4/CD26-positive signatures require careful validation (28, 29).

### 3.10 Integrated molecular network framework

The above pathways are best understood as a coupled network. Mechanical overload activates integrins, Piezo channels, MAPK and YAP/TAZ; mitochondrial dysfunction and altered metabolism reinforce ROS production; NF-kB and JAK/STAT increase cytokines and catabolic enzymes; impaired autophagy allows damaged organelles and inflammasome components to accumulate; and pyroptosis, ferroptosis or senescence releases additional danger signals. The transition from acute stress to chronic OA inflammation can therefore be summarized as: mechanical overload -> mechanotransduction -> mitochondrial dysfunction -> ROS and DAMP release -> NF-kB/MAPK/inflammasome activation -> immune-cell activation -> metabolic reprogramming -> senescence and SASP -> chronic inflammatory ecosystem. This framework explains why single cytokine inhibition often fails and why ecosystem-guided treatment should map a patient's dominant abnormality to the appropriate intervention level: edge, node, boundary condition or emergent state.

### 3.11 Evidence hierarchy and mechanistic caution

The molecular mechanisms summarized above are supported by different levels of evidence. In this review, human evidence was graded as follows. High: detection or functional association in human OA tissue, synovial fluid, blood or imaging-linked cohorts plus consistent support from *ex vivo*, genetic, interventional or independent validation studies. High-moderate: consistent human tissue or fluid association but fewer independent validation studies, more compartment-specific findings, or more mixed results across disease stages than pathways graded High. Moderate: consistent human tissue or fluid association, but limited functional validation, small cohorts or mixed results across studies. Low: predominantly animal-model or cell-culture evidence with sparse, indirect or conflicting human data. “No/experimental” indicates that clinical relevance remains hypothetical despite mechanistic plausibility. This grading is intended to make the evidentiary boundary transparent, not to imply formal systematic-review certainty.

Evidence levels were assigned through expert narrative assessment rather than through a formal GRADE or systematic-review procedure. Two authors independently reviewed the human and preclinical support for each major mechanism and resolved discrepancies by consensus. When direct human interventional evidence demonstrating OA-specific clinical benefit was lacking, the grade was intentionally conservative even if human tissue and mechanistic evidence were substantial. This clarification is particularly relevant for broadly studied signaling hubs such as NF-kB, where human OA tissue and experimental evidence are substantial but direct human OA therapeutic validation remains insufficient for the highest evidence category.

A rigorous mechanistic review should therefore separate association, pathway activation, genetic or pharmacological causality and clinical targetability. For example, detection of NLRP3 components suggests inflammatory potential, but experimental setting-specific negative evidence means that NLRP3 is not universally required. Similarly, mitochondrial ROS are repeatedly observed, but antioxidant trials require attention to delivery, timing and physiological redox signaling. This evidence hierarchy does not weaken the ecosystem framework; it strengthens it by identifying which nodes are mature therapeutic targets and which remain hypothesis-generating. Tables 2, 3 summarize the level of human support for major cellular and molecular modules to make these distinctions explicit.

**Table 2:** Cellular components of the OA inflammatory ecosystem and human evidence level.

Cellular component	Major signals or mediators	Pathological role	Therapeutic opportunity	Human evidence level
Synovial macrophages	IL-1beta, TNF-alpha, IL-6, CCL2, ROS, prostaglandins	Synovitis, immune recruitment, chondrocyte catabolism	Disease-associated macrophage-state reprogramming	High: human synovium/fluid plus models
Synovial fibroblasts	IL-6, CCL2, CXCL8, CXCL12, MMPs, growth factors	Inflammatory amplification, fibrosis, immune-cell organization	Target pathogenic stromal subsets and fibroblast-macrophage crosstalk	Moderate: human scRNA/tissue association
Chondrocytes	MMP-13, ADAMTS-4/5, NO, PGE2, SASP factors	Matrix degradation, hypertrophic-like transition, senescence	Restore anabolic-catabolic balance and inhibit pathological state transitions	High: human cartilage plus models
T cells	IL-17A, IFN-gamma, IL-10, TGF-beta	Context-dependent immune modulation	Endotype-specific modulation of adaptive immune balance	Low-moderate: subset evidence; context-dependent
Neutrophils and complement	NETs, proteases, C3a, C5a, membrane attack complex	Innate immune amplification and tissue injury	Biomarker discovery and selective innate pathway control	Low for NETs; moderate for complement
Endothelial and osteochondral cells	VEGF, PDGF-BB, RANKL/OPG, TGF-beta, Wnt signals	Angiogenesis, nerve ingrowth, bone remodeling and pain	Target osteochondral communication and vascular remodeling	Moderate: imaging/tissue plus models
Senescent cells	IL-6, IL-8, chemokines, MMPs, SASP programs	Inflammatory memory and impaired repair	Local senolytic or senomorphic therapy	Moderate: human markers; causality mostly models

Note for Tables 2, 3. Human evidence level follows the criteria stated in Section 3.11: High indicates human tissue/fluid or imaging-linked association plus functional or independent validation; High-moderate indicates consistent human association but fewer independent validations or more mixed compartment/stage-specific results than High; Moderate indicates consistent human association with limited functional validation or mixed results; Low indicates mainly preclinical or sparse/conflicting human data; No/experimental indicates hypothesis-generating evidence without established human clinical relevance.

**Table 3:** Molecular mechanisms regulating OA inflammatory persistence and human evidence level.

Pathway	Main triggers	Key effects	Therapeutic implication	Human evidence level
NF-kB	Cytokines, DAMPs, TLRs, ROS, mechanical stress	Cytokines, chemokines, COX-2, iNOS, MMPs, SASP	Local or cell-selective modulation preferred	High-moderate
NLRP3 inflammasome	ATP, ROS, mtDNA, ionic imbalance, crystals	IL-1beta/IL-18 maturation and pyroptotic amplification	Context-dependent endotype target	Moderate; context-dependent
MAPK/JAK-STAT	Cytokines, matrix fragments, oxidative stress	AP-1 and STAT-mediated inflammatory transcription	Requires precise modulation to preserve repair	High-moderate
Piezo/YAP/TAZ	Abnormal mechanical loading and matrix stiffness	Mechanosensitive calcium signaling and cell-state transitions	Target pathological mechanotransduction, not physiological loading	Moderate; stimulus-dependent
Mitochondria/autophagy	Aging, cytokines, metabolic stress, overload	ROS, DAMPs, impaired quality control	Restore physiological flux and mitochondrial homeostasis	High-moderate
Immunometabolism	Glycolysis, lactate, lipid and amino-acid changes	Stabilizes inflammatory macrophage and chondrocyte states	Metabolic reprogramming and biomarker selection	Moderate; stronger in models
Ferroptosis/pyroptosis/senescence	Lipid peroxidation, inflammasomes, DNA damage	Cell loss, DAMP release, SASP and inflammatory memory	Early-stage or phenotype-specific intervention	Low-moderate; mostly preclinical

## 4 Therapeutic strategies and translational implications

### 4.1 From inflammation suppression to ecosystem restoration

Therapeutic development in OA is shifting from generalized inflammation suppression toward restoration of immune and tissue homeostasis. The ecosystem framework makes treatment selection more explicit by linking diagnosis to intervention: ecosystem assessment identifies the disrupted level, endotyping identifies the dominant biological pattern, and therapy is selected according to whether the actionable target is an edge, a node, a boundary condition or an emergent state. Cytokine inhibitors mainly target communication edges; macrophage modulation targets cellular nodes; hydrogels and nanoparticles modify boundary conditions such as local retention and tissue penetration; senolytics and senomorphics target inflammatory memory; and MSCs or EVs modify paracrine repair and intercellular signaling. Table 4 illustrates this therapy-to-ecosystem mapping logic and highlights why a treatment can be mechanistically plausible yet clinically ineffective if it targets the wrong level, disease stage or endotype.

**Table 4:** Therapeutic approaches mapped to ecosystem target levels, clinical problems, current translational readiness and limitations.

Strategy	Ecosystem target level	Ecosystem problem addressed	Current translational readiness	Major limitation
NSAIDs/corticosteroids	Communication edge: prostaglandins and broad inflammatory transcription	Symptomatic inflammatory pain and effusion-synovitis flares	Clinically available symptomatic care	Limited structural disease modification and safety constraints
Cytokine inhibitors	Communication edge: IL-1, TNF or IL-6-related signaling	Single mediator-driven inflammatory amplification	Clinical evidence but limited OA efficacy	Network redundancy and poor patient selection
Macrophage-state modulation	Cellular node: synovial macrophage state	Pathological immune-cell activation and failed resolution	Early translational; no routine clinical use	Lack of validated human target-engagement markers
MSCs	Cellular node with paracrine edge effects: stromal-cell state and secretory signaling	Impaired repair and inflammatory imbalance	Early clinical investigation	Heterogeneous protocols; structural efficacy uncertain
Extracellular vesicles	Communication edge: intercellular protein/RNA/lipid cargo	Dysregulated cell-cell signaling and macrophage-chondrocyte crosstalk	Preclinical to early translational; no proven OA efficacy	Manufacturing, potency assays and human efficacy unresolved
Senolytics/senomorphics	Emergent state and node-state modifier: senescence, SASP and inflammatory memory	Inflammatory memory and failed repair resolution	Preclinical; experimental clinical relevance	Safety, specificity and target-engagement biomarkers
RNA therapeutics	Cellular node regulation: intracellular gene or ncRNA programs within disease-associated cells	Catabolic or inflammatory transcriptional programs	Preclinical only	Stability, intracellular delivery and cartilage penetration
Nanoparticles/hydrogels	Boundary condition: local retention, penetration and release kinetics	Poor intra-articular exposure and rapid clearance	Translational materials research; not routine care	Scale-up, repeat dosing and material safety

**Table 5:** Clinical lessons from inflammatory and regenerative OA studies.

Trial/approach	Target	Main lesson for OA inflammation
Anakinra	IL-1 receptor	Single IL-1 blockade was insufficient in unselected knee OA (61).
Lutikizumab	IL-1alpha/beta	Dual IL-1 inhibition produced limited benefit even in synovitis-enriched patients (62).
Etanercept	TNF pathway	OA differs from autoimmune arthritis; anti-TNF does not generalize well (63).
Canakinumab exploratory analysis	Systemic IL-1beta	Suggestive joint-replacement signal requires OA-specific confirmation (64).
MSC trials	Immune and trophic modulation	Symptom signals require standardization and structural validation (65–67).

**Table 6:** Candidate OA biological states, marker roles, therapeutic implications and clinical readiness.

Candidate biological state	Practical indicators and candidate biomarkers	Marker role in use	Therapeutic implications	Evidence maturity and clinical readiness
Inflammatory-dominant OA	MRI synovitis/effusion; high IL-6, CCL2, TNF-alpha or chemokine signals in serum/synovial fluid; macrophage/fibroblast signatures when available	Candidate biological endotype; MRI synovitis/effusion as imaging enrichment markers; inflammatory panels as candidate prognostic, predictive and target-engagement biomarkers, not validated treatment-selection cutoffs	Exploratory trial enrichment for macrophage-state modulation, pro-resolving biomaterials, locally retained anti-inflammatory approaches or selected cytokine-pathway strategies	Moderate maturity; suitable for exploratory trial enrichment, while treatment-selection thresholds and predictive biomarkers remain unvalidated.
Metabolic OA	High BMI, metabolic syndrome, insulin resistance, dyslipidemia; adipokines, mitochondrial or oxidative-stress markers in research settings	Clinical phenotype/boundary-condition profile; metabolic measures are practical risk and monitoring markers; mitochondrial or immunometabolic markers remain research biomarkers	Weight loss and exercise as clinical foundation; AMPK, mitochondrial protection or metabolic anti-inflammatory strategies as investigational add-ons	Emerging maturity. Clinical features such as obesity and metabolic syndrome are available, but validated mitochondrial or immunometabolic biomarkers are not yet routine.
Senescence-associated OA	Older age, prior injury, persistent low-grade inflammation; SASP markers such as IL-6/IL-8, p16INK4a/CDKN2A, DPP4/CD26 or EV cargo in research settings	Candidate biological endotype; SASP and senescence markers are mainly target-engagement or mechanistic biomarkers rather than routine prognostic or predictive tools	Local senomorphic or senolytic approaches, combined with repair-supportive or regenerative strategies	Experimental maturity. Biological rationale is strong, but SASP/senescent-cell burden markers, target-engagement assays and safety boundaries require validation.
Osteochondral-remodeling OA	Bone marrow lesions, subchondral sclerosis, osteophytes, angiogenesis/pain features; PDGF-BB, RANKL/OPG or bone-turnover markers in research settings	Structural phenotype and candidate biological state; MRI bone marrow lesions may serve as prognostic and endpoint markers; bone-turnover markers remain investigational	Osteochondral-unit targeting, timing-specific anti-resorptive/vascular modulation, regenerative repair or surgical strategies in advanced disease	Emerging to moderate maturity for imaging-based stratification. MRI bone marrow lesions and subchondral features are measurable, but linked targeted therapies need prospective testing.
Mechanical-structural dominant OA	Malalignment, instability, meniscal injury, focal cartilage defects, high-load occupation/sport exposure and relatively low inflammatory signal	Clinical phenotype and boundary-condition profile; alignment, instability and structural imaging are actionable clinical markers, not molecular endotypes	Biomechanical correction, exercise, weight management, orthoses, unloading, surgical evaluation when appropriate; biologics only if inflammatory or metabolic features coexist	Clinically established for biomechanical management. Molecular precision therapy is less mature; anti-inflammatory benefit may be limited unless inflammatory or metabolic features coexist.

### 4.2 Conventional anti-inflammatory therapy

NSAIDs remain important symptom-modifying drugs. They inhibit cyclooxygenase enzymes and reduce prostaglandin-mediated inflammatory pain (4, 5, 60). Topical NSAIDs can reduce systemic exposure, while oral NSAIDs provide stronger analgesia in selected patients. Nevertheless, they do not restore cartilage homeostasis or reverse the inflammatory ecosystem. Long-term use is limited by gastrointestinal, cardiovascular and renal risks, particularly in older patients. Intra-articular corticosteroids provide short-term relief, especially in effusion-synovitis phenotypes, but repeated use does not represent durable disease modification and must be weighed against potential structural and metabolic risks (4, 5).

### 4.3 Lessons from cytokine-targeted trials

Cytokine blockade has strong mechanistic rationale but inconsistent clinical performance. IL-1beta promotes NF-kB activation, MMP expression, ADAMTS activation and chondrocyte catabolism. However, intra-articular anakinra did not show significant symptomatic benefit compared with placebo in knee OA (61). Lutikizumab, a dual IL-1alpha/beta inhibitor, showed limited symptomatic improvement and no convincing suppression of synovitis in knee OA patients selected for synovitis (62). Etanercept failed to achieve robust primary pain efficacy in inflammatory erosive hand OA (63). An exploratory analysis of a cardiovascular trial suggested fewer hip or knee replacements with systemic IL-1beta inhibition, but this was not an OA-specific confirmatory trial (64). These results do not prove that inflammation is irrelevant. They show that unselected single-cytokine therapy is insufficient for a distributed inflammatory network. OA inflammation is redundant, compartmentalized and stage-dependent. Future cytokine studies should select patients using MRI synovitis, synovial fluid or serum biomarkers, tissue turnover markers and molecular endotypes rather than radiographic severity alone.

### 4.4 Macrophage-state modulation

Macrophages are attractive therapeutic targets because they coordinate both inflammatory activation and tissue repair. The desired approach is not macrophage depletion but reprogramming of pathological macrophage states toward resolution, efferocytosis and balanced remodeling. Potential tools include AMPK activation, PPARgamma-related pathways, mitochondrial modulators, nanoparticles, extracellular vesicles and immunomodulatory hydrogels. A major translational barrier is the lack of clinically validated macrophage-state biomarkers in synovial fluid, imaging or peripheral blood. Without such markers, it will be difficult to determine whether macrophage-directed therapy reaches its intended biological target in humans.

### 4.5 Mesenchymal stromal cells and extracellular vesicles

Mesenchymal stromal cells (MSCs) have been investigated because of their immunomodulatory and trophic properties. Early expectations that MSCs would directly differentiate into new cartilage have been tempered. The more defensible mechanism is paracrine regulation through growth factors, cytokines, immunoregulatory molecules and extracellular vesicles (63, 65–67). Small clinical trials using bone marrow-, adipose- or other tissue-derived MSCs have reported improvements in pain or function with acceptable safety, but protocols differ substantially in cell source, donor status, culture expansion, dose, injection schedule and outcome measures (63, 65, 66). Definitive evidence that MSCs regenerate cartilage or reverse OA in humans remains limited. Extracellular vesicles provide a cell-free alternative. MSC-derived EVs can deliver proteins, lipids and regulatory RNAs, modulate macrophage activation and support chondrocyte survival in preclinical models (67–69). Compared with live-cell therapy, EVs may offer easier storage and engineering. Nevertheless, no reproducible human efficacy dataset currently establishes EV therapy as a disease-modifying OA treatment; EVs should therefore be described as preclinical or early translational rather than clinically validated.

### 4.6 Senescence-targeted therapy and RNA therapeutics

Senolytics aim to eliminate selected senescent cells, whereas senomorphics suppress pathological SASP output without cell killing. Experimental evidence in post-traumatic OA supports senescent-cell clearance as a disease-modifying concept (27), and broader aging studies support the translational potential of senolytics (70–72). Yet senescent cells may participate in wound healing and tumor suppression, and systemic senolysis can have off-target risks. Local delivery, biomarker-confirmed target engagement and stage-specific treatment windows will be essential. RNA-based therapy offers gene-level control of inflammatory or catabolic pathways. miRNA mimics, antagomirs, siRNA and antisense oligonucleotides could target MMPs, cytokines, senescence regulators or metabolic enzymes. miR-140 restoration is a representative cartilage-protective concept (53, 54). However, RNA therapy for OA remains preclinical; no human efficacy data are available, and major barriers include nuclease degradation, intracellular delivery, cartilage penetration, off-target effects and repeat-dosing safety. RNA therapeutics should therefore be discussed as a platform requiring delivery-system validation rather than as a near-term clinical treatment.

### 4.7 Nanomedicine and local delivery systems

The joint is challenging for drug delivery. Small molecules are rapidly cleared from synovial fluid, cartilage is dense and avascular, and systemic exposure increases adverse effects. Nanoparticles, cartilage-binding ligands, polymeric carriers, lipid nanoparticles, injectable hydrogels and stimulus-responsive materials may improve joint retention, controlled release and tissue penetration (73–76). Local delivery is particularly relevant for NF-kB, NLRP3, senescence or RNA-based interventions, where systemic modulation could impair host defense or normal repair. The strongest future platforms are likely to combine functions: anti-inflammatory activity, antioxidant capacity, RNA or protein delivery, macrophage-state modulation and regenerative cues. However, materials must be evaluated for biodegradation, immunogenicity, reproducibility, scale-up and compatibility with repeated intra-articular administration. Nanomedicine should not be framed only as a delivery add-on; it may define whether mechanistic targets become clinically feasible.

### 4.8 Disease-modifying OA drugs and lessons beyond inflammation

Disease-modifying OA drug development has been difficult because OA progression is slow, heterogeneous and only partially captured by pain outcomes. Some structure-directed approaches have increased cartilage thickness or altered tissue biomarkers without producing parallel clinically meaningful pain improvement in the overall population. This dissociation illustrates that structural change and symptoms can diverge. Wnt-pathway modulation, catabolic enzyme inhibition and subchondral bone-directed strategies have also highlighted the need for precise patient selection and endpoints. These lessons are directly relevant to inflammation-targeted therapy. A drug that reduces synovitis may not regenerate cartilage; a regenerative therapy may not relieve pain if synovial inflammation or subchondral nerve ingrowth persists; and a structural drug may fail if tested in patients without measurable progression. Future trials should combine clinical outcomes, MRI-based cartilage and synovitis measures, bone marrow lesion assessment, biomarker changes and patient-reported outcomes. Disease modification should be defined as a coherent effect across biology, structure and symptoms, not a change in one domain alone.

### 4.9 Combination therapy and timing

Because OA is a network disease, combination therapy may be required. Examples include macrophage-state modulation plus mitochondrial protection, senomorphic therapy plus local matrix-preserving delivery, or MSC/EV-based regenerative signals combined with hydrogel-mediated retention. Mechanistically compatible combinations must be selected based on endotype and disease stage. Early inflammatory OA may benefit from inflammation-resolution strategies, intermediate OA from immune-metabolic modulation and tissue protection, and advanced OA from regenerative or surgical approaches. Timing may be as important as target selection. In early OA, mechanical and mitochondrial stress may still be reversible, chondrocytes retain greater plasticity and synovitis may be modifiable. In late OA, cartilage loss, osteophytes, fibrosis and osteochondral remodeling may be too advanced for anti-inflammatory therapy alone. Therefore, early molecular diagnosis is a central requirement for future disease modification. Biomarkers and imaging should identify active progression before irreversible structural failure.

### 4.10 Precision medicine implications

Box 2: Why single-target anti-inflammatory therapy is insufficient in OA. Why single-target anti-inflammatory therapy is insufficient in OA OA inflammation is heterogeneous across patients, joint compartments and disease stages. Inflammatory pathways are redundant; blocking IL-1beta does not eliminate TNF signaling, ROS production, mechanical stress responses or SASP-mediated inflammation. Drug delivery is limited by rapid synovial clearance, poor cartilage penetration and systemic safety concerns. Late-stage structural damage may be irreversible even when inflammation is reduced; early endotype-guided intervention is therefore critical. For Frontiers in Medicine readers, the central therapeutic implication is not that every OA patient requires a new immunotherapy. Rather, the ecosystem framework helps define when immune or metabolic intervention is biologically plausible and when mechanical, structural or surgical management is more appropriate. Inflammatory-dominant OA, when defined by prespecified MRI synovitis/effusion and inflammatory-marker criteria, may be the most feasible candidate state for exploratory immune-modulation trials, but it should not yet be treated as a validated predictive endotype. Metabolic OA is clinically recognizable through obesity, insulin resistance and systemic metabolic risk, but joint-specific metabolic biomarkers remain immature. Senescence-associated OA is conceptually important but still experimental because senescent-cell burden and SASP activity are not routinely measurable. Osteochondral-remodeling OA and mechanical-structural OA are already clinically actionable through imaging, biomechanical assessment and surgery when indicated. Precision medicine should therefore be interpreted as staged enrichment rather than immediate individualized molecular therapy. The first aim is to avoid biological dilution in clinical trials by selecting patients whose ecosystem state matches the intervention. The second aim is to choose endpoints that correspond to the treatment mechanism: synovitis measures for anti-inflammatory therapy, structural MRI for disease-modifying approaches, metabolic measures for metabolic interventions, and pain and safety outcomes for analgesic strategies. This practical interpretation is more realistic than assuming that omics-based classification will soon be available for routine OA care.

### 4.11 Ecosystem mismatch as a clinical failure mechanism

The mixed performance of OA therapeutics should be interpreted not only as a list of positive and negative agents, but as evidence for ecosystem mismatch. Target engagement must be demonstrated in the joint compartment relevant to the mechanism; patient selection must match the dominant ecosystem state; and endpoints must match the expected biological effect. A systemic biomarker change may not mean that synovial inflammation, cartilage biology or osteochondral remodeling has been modified. Four recurrent mismatches are especially important. A target-level mismatch occurs when a therapy suppresses a communication edge while the dominant pathology resides in a cellular node or boundary condition. IL-1 or TNF blockade can reduce selected mediators but may not correct persistent macrophage-fibroblast states, mitochondrial stress, abnormal loading or senescence-associated inflammatory memory. This helps explain the limited efficacy of single-cytokine strategies in unselected OA despite strong mechanistic rationale (61–64). A population mismatch occurs when symptomatic radiographic OA is used as the main eligibility criterion. A cytokine inhibitor tested in patients without synovitis-dominant inflammation, an MSC product tested without evidence of reparative failure, or a senolytic tested without measurable senescence burden is likely to generate a neutral average effect. Such results may indicate poor biological matching rather than invalidity of the target itself (77–80). A timing mismatch occurs when treatment is introduced after the modifiable phase has passed. In early or intermediate OA, synovitis, macrophage activation, mitochondrial stress and catabolic chondrocyte states may remain plastic; in advanced OA, cartilage loss, osteophytes, fibrosis and osteochondral remodeling may dominate. An endpoint mismatch occurs when the outcome does not reflect the mechanism: pain-targeted therapies require symptom and safety monitoring, whereas structure-targeted or delivery-based strategies require target engagement, retention, imaging and tissue-specific readouts (81–86). The clinical message is that therapies should be tested in the ecosystem context in which their mechanism is plausible, and combination treatment should be rational and endotype-matched rather than empiric.

### 4.12 Safety considerations in immune ecosystem manipulation

Immune modulation in OA must preserve protective functions. NF-kB, JAK/STAT, macrophages, autophagy and senescence are not purely pathological; they contribute to host defense, debris clearance, tissue remodeling and tumor suppression. Over-suppression could impair repair or increase susceptibility to infection. The desired endpoint is resolution of chronic pathological inflammation, not immune paralysis. Local delivery, intermittent dosing, target-specific release and biomarker-guided discontinuation may improve the therapeutic index.

## 5 Challenges and future perspectives

### 5.1 Biological heterogeneity and inflammatory endotypes

A central translational barrier is heterogeneity. Radiographic grade, pain score and joint location do not adequately reflect molecular mechanisms. Endotype discovery using biochemical markers has identified subgroups related to low tissue turnover, structural damage and systemic inflammation (74). Future classification should integrate clinical features, MRI synovitis and effusion, bone marrow lesions, cartilage composition, circulating and synovial biomarkers, metabolic profiles and, when available, transcriptomic signatures. Inflammatory-dominant OA may show macrophage-fibroblast activation and high cytokine/chemokine signals; metabolic OA may be associated with obesity, dyslipidemia and mitochondrial stress; senescence-associated OA may show SASP and aging-related markers; mechanical-dominant OA may be driven by malalignment, instability or focal overload. These should be interpreted as partially overlapping candidate states rather than fixed, mutually exclusive diseases. Their value depends on whether they can be measured reproducibly and whether they predict progression or treatment response better than conventional clinical and radiographic variables. Biological heterogeneity also explains why apparently rational therapies can fail in unselected trials. A treatment that suppresses IL-1 signaling may show little average effect if only a minority of enrolled patients have IL-1-dominant synovial inflammation. A regenerative or trophic therapy may appear inconsistent if some participants have advanced osteochondral remodeling, others have inflammatory synovitis and others have pain driven mainly by malalignment or central sensitization. In this setting, a negative trial may reflect biological dilution rather than absence of target relevance. The ecosystem framework therefore treats endotypes as dominant network states rather than mutually exclusive diseases. Inflammatory, metabolic, senescence-associated, osteochondral-remodeling and mechanical-structural patterns can overlap within the same joint and change over time. Early OA may be dominated by mechanosensing, mitochondrial stress and innate immune activation; intermediate OA may show macrophage-fibroblast amplification and cartilage matrix degradation; advanced disease may be characterized by fibrosis, senescence, osteophytes and altered subchondral bone. A single patient can therefore move between states as the joint environment changes. For clinical translation, the most useful candidate endotype will not necessarily be the most molecularly precise one. It will be the category that can be identified reproducibly, predicts progression or treatment response and can be linked to a feasible intervention. MRI-defined synovitis is closer to trial readiness than synovial single-cell profiling; BMI and metabolic syndrome are more clinically accessible than mitochondrial proteomics; and mechanical malalignment is more actionable in routine care than most molecular signatures. The purpose of endotyping is thus pragmatic: to reduce biological mismatching and make trials more interpretable, while allowing more advanced omics to refine mechanisms in selected cohorts.

### 5.2 Biomarkers and multi-omics

Useful biomarkers should identify disease activity, predict progression, select treatment and monitor response. Candidate inflammatory markers include IL-6, CCL2, TNF-alpha, IL-17 and chemokines; tissue turnover markers include COMP, CTX-II and aggrecan fragments; EV cargo and circulating miRNAs may provide additional information. Single biomarkers are unlikely to be sufficient because OA inflammation is spatially heterogeneous and fluctuates over time. Multi-marker panels combined with imaging and clinical variables are more plausible. Table 7 summarizes the main human evidence supporting the ecosystem components most relevant to clinical translation. Single-cell and spatial multi-omics are redefining OA biology. Cartilage scRNA-seq has identified distinct chondrocyte states during OA progression (24), and synovial scRNA-seq has revealed cellular heterogeneity in OA synovium (25). Spatial transcriptomics, proteomics and metabolomics can preserve tissue localization and map ligand-receptor interactions among macrophages, fibroblasts, chondrocytes, endothelial cells and subchondral bone cells. The next step is to move from descriptive cell clusters to actionable cellular neighborhoods that predict progression or treatment response.

**Table 7:** Human evidence supporting major components of the OA inflammatory ecosystem, evidence maturity and current limitations.

Ecosystem component	Representative human evidence	Clinical or translational relevance	Major limitations of current human evidence	Evidence maturity for clinical use
Synovitis and effusion	MRI studies, synovial tissue analyses and synovial-fluid inflammatory mediators support clinically meaningful synovial involvement in subsets of OA.	Supports inflammatory-dominant trial enrichment and monitoring of treatment engagement.	Thresholds for treatment selection are not standardized; synovitis may be a driver, amplifier or consequence depending on stage and compartment.	Established for clinical relevance
Macrophage and fibroblast node states	Human OA synovial single-cell and tissue studies identify heterogeneous immune and stromal populations.	Provides rationale for node-focused immunomodulation and stromal-immune targeting.	Most evidence is cross-sectional; causality, accessible surrogate markers and durable phenotype modulation remain uncertain.	Emerging; mainly tissue/scRNA evidence
Chondrocyte state transitions and senescence	Human OA cartilage studies show catabolic, hypertrophic-like, inflammatory and senescence-associated signatures, especially in advanced tissue.	Supports biomarkers of cartilage stress and inflammatory memory.	Many samples are end-stage surgical tissues; early disease dynamics and causal links require longitudinal validation.	Moderate for tissue association; early for treatment selection
Osteochondral remodeling and pain-related structures	MRI bone marrow lesions, subchondral bone changes and osteochondral tissue studies link bone-cartilage remodeling with symptoms and progression.	Supports osteochondral-remodeling endotypes and structural endpoint selection.	Mechanistic drivers vary by stage, joint compartment and loading context; structural-pain relationships are imperfect.	Moderate for structural association
Metabolic and adipose-related boundary conditions	Human epidemiology, obesity-associated OA studies, adipokine data and metabolic profiling support systemic and local metabolic contributions.	Supports metabolic-risk assessment and non-pharmacologic intervention as part of ecosystem management.	Mechanical load, adiposity and systemic metabolic inflammation are difficult to separate in many human cohorts.	Moderate for clinical association; mechanistic markers emerging
Multivariable biomarkers and endotype signatures	Biochemical clustering, FNIH/OAI-type biomarker-imaging analyses and multi-marker approaches suggest biological subgroups beyond radiographic grade.	Supports trial enrichment and progression-risk modeling.	Prospective treatment-selection trials are limited; clinical utility beyond radiographic grade and symptoms remains to be proven.	Emerging; not yet routine clinical stratification

### 5.3 AI-assisted precision medicine and human-relevant models

Artificial intelligence may help integrate radiographs, MRI, clinical features, patient-reported outcomes, biomarkers and omics data. Applications include OA subtype classification, rapid progression prediction, treatment-response modeling and clinical-trial enrichment (77, 78). However, AI models require large standardized datasets, external validation and interpretability. They should support, not replace, biological reasoning. Human-relevant models are also needed. Surgically induced and chemically induced animal models are invaluable but do not fully reproduce decades-long human OA, human cartilage thickness, metabolic comorbidity or clinical pain. Cartilage-on-chip and joint-on-chip models can integrate mechanical loading, inflammatory stimuli and human cell interactions (87). Patient-derived explants, organoids and multi-tissue co-cultures may bridge the gap between reductionist cell culture and animal models.

### 5.4 Future clinical trial framework

Future OA trials should move from broad radiographic inclusion to biological enrichment. A precision-oriented trial could proceed in four stages. First, patients are phenotyped using clinical features, BMI, pain characteristics, disease duration, previous injury, alignment, joint distribution and baseline radiographic severity. Second, imaging identifies synovitis, effusion, bone marrow lesions, cartilage defects, osteophytes and meniscal or ligamentous structural abnormalities. Third, targeted molecular assays evaluate inflammatory mediators, tissue turnover, EV cargo, metabolic markers and senescence-related signals. Fourth, patients are assigned to mechanism-matched interventions according to the dominant ecosystem abnormality: cytokine or macrophage modulation for inflammatory-dominant states, mitochondrial or AMPK-targeted therapy for metabolic states, senescence-targeted therapy for aging-associated states and regenerative, biomechanical or structural intervention for advanced degeneration.

For inflammatory-dominant OA, a practical trial definition should remain explicit and conservative. A candidate inflammatory-dominant state could be defined as symptomatic OA with prespecified MRI synovitis and/or effusion, supportive inflammatory biomarker evidence in serum or synovial fluid, and no clearly dominant alternative explanation such as severe malalignment, gross instability or end-stage structural collapse. This definition should be treated as an enrichment rule, not as a validated diagnostic label.

Primary and secondary endpoints should match the intervention mechanism. For immune-modulating therapies, primary endpoints may include WOMAC pain or function at 6 months, with key secondary endpoints including MRI synovitis/effusion, inflammatory-marker changes, target-engagement readouts and durability at 12 months. For structure-directed therapies, MRI cartilage thickness, bone marrow lesion change and radiographic progression may be more appropriate. For metabolic interventions, weight, insulin resistance, lipid-related measures and metabolic-inflammatory biomarkers should be incorporated. Safety and rescue analgesic use should be prespecified across all trial types.

Changing endotypes over time should be handled prospectively rather than ignored. Baseline NEBE assignment should be repeated at prespecified follow-up intervals, and analyses should treat ecosystem state as a time-dependent variable when appropriate. Patients who shift from inflammatory to mechanical-structural dominance, or from metabolic stress to osteochondral remodeling, may require different endpoint interpretation or treatment reassignment in adaptive trial designs. This approach directly tests whether ecosystem-guided stratification adds value beyond conventional radiographic inclusion.

### 5.5 Clinical implementation without universal omics

A clinically feasible implementation strategy will likely rely on stepwise stratification rather than universal multi-omics profiling. In routine practice, most patients can be assessed using age, BMI, comorbid metabolic disease, pain phenotype, joint distribution, alignment, radiographs and, when available, MRI findings such as synovitis, effusion, bone marrow lesions and structural damage. This level is sufficient to identify mechanical-structural disease, likely metabolic contribution, end-stage structural disease and patients who may require referral or imaging-based stratification. In specialty clinics and clinical trials, additional markers can be added selectively. These may include inflammatory proteins, tissue-turnover markers, synovial-fluid analysis, metabolic measures or imaging-based progression features. High-cost approaches such as single-cell sequencing, spatial transcriptomics, proteomics and AI-derived multi-omics signatures should primarily support discovery, difficult-case characterization and trial enrichment rather than routine care. The practical goal is not universal omics, but clinically feasible enrichment that reduces false-negative trials caused by biological mismatching.

### 5.6 Regulatory and manufacturing challenges

Regenerative and delivery-based therapies introduce regulatory challenges that differ from small molecules. MSCs require standardization of donor eligibility, tissue source, culture conditions, passage number, viability, potency and release criteria. EVs require definitions of particle identity, cargo, purity, potency and storage stability. Nanoparticles and hydrogels require reproducible manufacturing, degradation and local safety testing. Without these standards, apparently promising preclinical results may not translate into comparable clinical products. Outcome standardization is equally important. Trials should predefine responder criteria, structural endpoints, biomarker endpoints and safety monitoring. Pain alone is insufficient because pain is influenced by central sensitization, mood, comorbidity and expectations. Structural endpoints alone are also insufficient because cartilage thickness does not always correlate with symptoms. A credible DMOAD trial should include a prespecified biological target-engagement readout, a structural measure and a patient-centered outcome.

### 5.7 Outstanding questions

Key unresolved questions remain. Which cellular populations are causal drivers rather than consequences of OA? Can inflammatory phenotypes be detected before irreversible structural damage? Which biomarkers predict response to cytokine, macrophage, metabolic, senescence or regenerative therapies? Can inflammation be resolved without impairing tissue repair? What delivery systems can reach synovium, cartilage and osteochondral compartments reproducibly? Addressing these questions will require longitudinal cohorts, spatial multi-omics, human-relevant models, standardized biomarker platforms and mechanism-informed trial design.

### 5.8 Proposed roadmap for precision OA trials

A feasible precision-trial roadmap would begin with biological enrichment. Candidate participants would be screened for symptomatic and radiographic OA, then stratified by MRI synovitis, effusion, bone marrow lesions, cartilage defects, BMI, metabolic markers and inflammatory biomarkers. Participants with inflammatory-dominant candidate states could be considered for exploratory trials of macrophage modulation, cytokine regulation or pro-resolving biomaterials. Those with metabolic OA could enter mitochondrial, AMPK or weight-loss-plus-pharmacology studies. Those with senescence-associated signatures could enter local senomorphic or senolytic studies. Structural-dominant phenotypes could be assigned to regenerative, osteochondral-unit or biomechanical interventions.

Worked example: how NEBE may change trial inclusion. A 62-year-old patient with KL grade 3 knee OA, persistent pain, MRI-confirmed synovitis and effusion, elevated IL-6/CCL2-type inflammatory signals and obesity-associated metabolic stress would traditionally be classified as symptomatic radiographic knee OA. Under the NEBE framework, this patient would be described as having macrophage/stromal-node activation, inflammatory cytokine/chemokine edges, obesity-associated metabolic boundary stress and an emergent state of persistent synovitis with inflammatory pain. This patient may be suitable for an exploratory trial testing local immune-ecosystem modulation with synovitis and inflammatory-marker target-engagement endpoints. By contrast, another KL grade 3 patient with focal cartilage loss, marked malalignment, minimal synovitis and low inflammatory biomarkers would not be enriched for the same immune-modulating trial, even if pain severity and radiographic grade were similar. The practical change is not routine individualized molecular therapy, but more mechanism-consistent trial inclusion, target selection and endpoint matching.

The trial should include target-engagement measures. For a macrophage therapy, synovial fluid cytokines, macrophage-related EV cargo or imaging of synovitis could be used. For a mitochondrial therapy, oxidative stress markers, metabolic profiles and cartilage composition imaging may be relevant. For a senescence strategy, SASP markers and senescence-associated cell-surface markers could be followed. Such designs would make negative trials more interpretable: if a drug fails despite clear target engagement, the biological target may be wrong; if it fails without target engagement, the delivery or dose may be inadequate.

### 5.9 Ecosystem validation roadmap

A framework-driven review should specify how its organizing logic can be validated. The proposed ecosystem framework can be tested through linked discovery, validation and intervention cohorts rather than through a single cross-sectional omics study. Discovery cohorts should use human OA tissues, synovial fluid, serum, MRI and clinical outcomes to define reproducible cellular nodes, communication edges and boundary conditions. Single-cell and spatial tools are useful at this stage, but findings should be translated into clinically measurable imaging or biomarker surrogates (88–90). Longitudinal validation cohorts should test whether combinations of nodes, edges and boundary conditions predict clinically meaningful outcomes better than single markers or radiographic grade alone. Outcomes should include pain trajectories, MRI synovitis, cartilage thickness loss, bone marrow lesion development, radiographic progression and treatment escalation. Models should be evaluated for discrimination, calibration, decision-curve benefit and external validation, following the logic of imaging and biomarker programs such as the FNIH OA Biomarkers Consortium (80, 91–94). Interventional cohorts are the decisive test. Patients should be assigned to therapies matched to their dominant ecosystem abnormality and prespecified target-engagement readouts. A cytokine trial should show suppression of the intended inflammatory edge, a macrophage therapy should show node-state reprogramming, a metabolic therapy should show mitochondrial or oxidative target engagement, and a delivery platform should show local retention or tissue exposure. Negative results would then be interpretable as wrong target selection, inadequate delivery, insufficient enrichment or failure of the ecosystem assumption itself. This roadmap also clarifies the role of omics. Omics should not be presented as a routine clinical requirement before there is evidence of utility. Instead, it should be used to discover and validate simpler biomarker panels, imaging signatures and decision rules. The framework becomes clinically valuable only if it improves stratification, predicts response or explains failure in ways that change trial design or patient management (95–113).

### 5.10 Testable predictions derived from the ecosystem framework

The ecosystem framework should be falsifiable rather than purely descriptive. Three predictions follow directly from the node-edge-boundary framework proposed in this review.

Prediction 1. In patients with inflammatory-dominant OA, therapies that reprogram disease-associated macrophage or stromal-cell states should produce more durable clinical and biological improvement than blockade of a single cytokine. A testable design would compare a node-targeted strategy, such as local macrophage-state modulation or pro-resolving biomaterial delivery, with an edge-targeted strategy, such as IL-1beta blockade, in MRI- and biomarker-confirmed synovitis-enriched OA. The prediction does not require a universal effect-size threshold *a priori*; rather, it would be supported if the node-targeted arm shows a prespecified clinically meaningful advantage in WOMAC pain or function, MRI synovitis/effusion and multi-marker inflammatory signatures at 6 months with persistence at 12 months. Failure to show meaningful advantage despite verified target engagement would argue against the practical relevance of macrophage/stromal-node dominance in that endotype. Prediction 2. Patients with simultaneous abnormality across multiple ecosystem nodes and boundary conditions, such as synovitis, cartilage degradation, osteochondral remodeling, obesity-associated metabolic stress and abnormal loading, should progress faster than patients with isolated single-compartment abnormality. This can be tested in longitudinal cohorts using predefined composite network-burden scores derived from MRI, soluble biomarkers and metabolic profiling. Support would require the composite score to improve time-to-event or longitudinal models for cartilage loss, incident bone marrow lesions, worsening pain, radiographic progression or joint replacement over single-component models, with effect estimates robust to adjustment for age, sex, BMI, analgesic use and baseline radiographic grade. If a single variable performs equivalently across independent cohorts, the ecosystem framework would add limited prognostic value. Prediction 3. Prognostic or treatment-response models based on multivariable network features, including cytokines, chemokines, metabolites, EV cargo, MRI synovitis, bone marrow lesions and loading-related variables, should outperform models based on any single mediator. This prediction can be evaluated using prespecified model-comparison metrics, such as cross-validated area under the receiver-operating characteristic curve, calibration slope, decision-curve net benefit, net reclassification improvement or clinically meaningful reclassification across independent validation cohorts. Statistically significant but clinically negligible changes, such as a very small Delta AUC without improved calibration or decision-curve benefit, would not constitute strong support for the practical utility of the ecosystem framework. If these predictions are not supported in independent human datasets, the ecosystem framework should be revised or abandoned in favor of a simpler disease explanation.

### 5.11 Limitations and future validation of the ecosystem framework

Several limitations should be acknowledged. First, the ecosystem framework is assembled from heterogeneous evidence streams, including human tissue studies, imaging-linked cohorts, biomarker studies, cell culture, animal models and early clinical trials. These sources differ in disease stage, tissue compartment, sampling method and outcome definition. Second, many causal links are not yet proven in prospective human OA cohorts; single-cell and spatial studies identify candidate nodes and edges, but they do not by themselves establish which interactions drive progression or treatment response. Third, proposed endotypes remain provisional working categories rather than validated diagnostic labels, and should be presented as candidate biological states until prospective reproducibility, stability and treatment-response prediction are demonstrated. Their clinical value depends on whether they can be identified reproducibly using feasible markers and whether they predict progression, target engagement or therapeutic response better than radiographic grade alone. Fourth, biomarkers, extracellular vesicle assays, metabolomics and omics-derived signatures require standardization before routine use. Fifth, this review emphasizes inflammation and immunometabolism, but OA remains strongly shaped by mechanical loading, structural damage, rehabilitation, weight management and surgical decision-making. The framework should therefore be tested as a tool for stratification and trial design, not as a replacement for established clinical assessment. Future validation should prioritize longitudinal human cohorts, independent biomarker replication, imaging-linked progression studies and endotype-enriched interventional trials.

## 6 Conclusion

OA is a heterogeneous whole-joint disorder in which chronic low-grade inflammation interacts with mechanical loading, metabolic stress, senescence and impaired repair. The inflammatory microenvironment can be conceptualized as an immune-metabolic ecosystem rather than a list of cytokines. Macrophages, fibroblasts, chondrocytes, endothelial cells and osteochondral cells form reciprocal communication networks regulated by mechanotransduction, mitochondrial stress, NF-kB/MAPK/JAK-STAT signaling, inflammasomes, autophagy, immunometabolism and senescence-associated inflammatory memory. The limited efficacy of unselected single-cytokine therapy indicates that future disease-modifying strategies will likely need to be state-guided, locally deliverable and capable of restoring joint homeostasis.

The NEBE framework does not claim to discover new molecular components of OA and should not be interpreted as an established clinical classification system. Its contribution is organizational and hypothesis-generating: it converts established mechanisms into a testable node-edge-boundary-emergent framework that generates expectations about network-level progression, state-matched responses and multi-node therapeutic vulnerability. Ultimately, the framework should be judged by whether it improves patient stratification, predicts treatment response and guides prospective clinical trial design, not by whether it names additional molecular targets.

Its clinical value should therefore be tested in prospective human cohorts and biomarker-guided trials assessing whether network-based stratification improves prognosis, target selection, target engagement or treatment response beyond single-pathway, radiographic or anatomy-only frameworks. A temporal perspective is essential: ecosystem-guided therapy will be most useful if it can identify when inflammatory networks remain modifiable and when structural boundary conditions dominate clinical outcomes.

## Data Availability

No original datasets were generated or analyzed for this review. All sources discussed in the article are cited in the reference list.
